# Epidemiology, aetiology and clinical characteristics of clostridial bacteraemia: a 6-year population-based observational study of 386 patients

**DOI:** 10.1007/s10096-022-04491-8

**Published:** 2022-09-22

**Authors:** Maaria Sarasoja, Bo Nilson, Daniel Wide, Åsa Lindberg, Gustav Torisson, Karin Holm

**Affiliations:** 1 Department of Clinical Sciences, Division of Infection Medicine, Lund University, BMC B14, Tornavägen 10, 22184 Lund, Sweden; 2 Infectious Diseases, Skåne University Hospital, Malmö, Sweden; 3 Infectious Diseases, Skåne University Hospital, Lund, Sweden; 4 Department of Clinical Microbiology, Infection Control and Prevention, Office for Medical Services, Region Skåne, Lund, Sweden; 5 Department of Laboratory Medicine Lund, Division of Medical Microbiology, Lund University, Lund, Sweden; 6 Halland Hospital, Halmstad, Sweden; 7 Department of Translational Medicine, Division of Clinical Infection Medicine, Lund University, Malmö, Sweden

**Keywords:** Clostridium, Bacteraemia, Blood stream infection, Incidence, Sepsis, Antibiotic susceptibility

## Abstract

The objective of this study is to provide a population-based clinical, epidemiological and microbiological overview of clostridial bacteraemia. All cases of bacteraemia in the Skåne Region between 2014 and 2019 with a species currently belonging to the Clostridium genus were identified in the regional clinical microbiology database. Clinical data were retrieved by medical chart-review. A total of 386 unique episodes of clostridial bacteraemia were found resulting in an incidence rate of 4.9/100.000 person-years. The median age was 76 with 56% males. The incidence rate ratio was 34.3 for those aged 80 + vs 0–59. The minimum inhibitory concentrations varied between species but were universally low for metronidazole and carbapenems. Malignancy was the most common co-morbidity, in 47% of patients and most pronounced for C. septicum. Criteria for sepsis and septic shock were met in 69% and 17%, respectively. The 28-day mortality was 26%. High age, absence of fever, high C-reactive protein and high SOFA-score were all significantly associated with mortality. We present the highest incidence rate of clostridial bacteraemia to date. Clostridial bacteraemia is a severe condition with acute onset, affecting elderly with co-morbidities, most pronounced malignancies. Mortality is related to acute manifestations rather than to background factors.

**Supplementary information:**

The online version contains supplementary material available at 10.1007/s10096-022-04491-8.

## Introduction

*Clostridium* is a heterogenous group of Gram-positive anaerobic bacteria causing severe soft tissue infections including gas gangrene, intra-abdominal infections and clinical conditions such as botulism and tetanus [1, 2]. Clostridial bacteraemia is considered relatively uncommon and accounts for 1–3% of all invasive bacteraemia in hospitalised patients, *C. perfringens* being the most commonly identified species [1, 3, 4, 5, 6]. Notably, many species formerly within the *Clostridium* genus have been re-classified making comparisons between studies difficult. Clostridial bacteraemia often affects compromised hosts such as patients with diabetes or underlying malignancies [7, 8]. The correlation between *C. septicum* and malignancies is particularly well established, and studies have showed that up to 80% of episodes with *C. septicum* bacteraemia are associated with an underlying malignancy [9, 10, 11, 12]. Several case reports of clostridial bacteraemia describe massive haemolysis with rapid fatal outcome which has been estimated to occur in 7–15% of *C. perfringens* bacteraemia [13, 14, 15, 16]. Moreover, estimates of mortality in clostridial bacteraemia vary between 17 and 60% depending on species and study population [3, 17, 18, 19]. However, cases with asymptomatic clostridial bacteraemia have also been reported [20], and since the frequency of severe manifestations and fatal outcome is mostly estimated from case reviews and single centre studies, the true frequency remains largely unknown. The so far largest, and to our knowledge only, population-based study from Calgary, Canada, presents an incidence of 1.8/100.000/year for clostridial bacteraemia and a mortality of 30% but does not distinguish between clinical manifestations [8].

The aims of the present study were to describe population-based epidemiological and microbiological data on clostridial bacteraemia including data on antibiotic susceptibility. Moreover, we aimed to describe clinical spectrum in relation to clostridial species and factors associated with mortality and provide more accurate data on the frequency of massive haemolysis in *C. perfringens* bacteraemia.

## Methods

### Study design and setting

This is a retrospective population-based observational study on clostridial bacteraemia in the Skåne region, Sweden, between January 2014 and December 2019.

Skåne is a region in the southernmost part of Sweden, with 1.4 million inhabitants (2019). There are ten hospitals dispersed throughout the Skåne region, nine public and one privately run, all using the same electronical medical system. Almost all aspects of modern in-patient healthcare are provided by these hospitals. Thus, the absolute majority of the population of Skåne would seek healthcare in the region.

### Identification of participants and microbiological data analyses

Case-finding was performed retrospectively through the laboratory databases of Department of Clinical Microbiology, Region Skåne, Lund, Sweden, the sole service-provider of microbiological diagnostics for the region. All blood cultures with a positive finding within the *Clostridium* genus were extracted from the electronical laboratory database in January 2020. Individuals were identified by their unique personal identity number and in case of multiple bacteraemic episodes with *Clostridium*, a 3-month deduplication period was employed.

Regional policy during this time period stated that two complete sets (aerobic/anaerobic bottles) should be drawn from separate venepunctures when a bloodstream infection is suspected. From January 1st 2014 to December 31st 2014, the BacT/ALERT® blood culture system was used with the bottles BacT/ALERT FA Plus and BacT/ALERT FN Plus (bioMérieux, Inc.). From January 1st 2015, the blood culture system was changed to BACTEC™ FX with the bottles BACTEC Plus Aerobic, and BACTEC Lytic Anaerobic or BACTEC Plus Anaerobic (BectonDickinson) depending on what bottles were available. Cultures were incubated for 120 h before regarded as negative. The five largest hospitals in the region have on-site blood culturing systems for local incubation, and upon positive signalling, the bottle was transferred to the Department of Clinical Microbiology where species determination was performed with Microflex matrix–assisted laser desorption ionisation-time of flight mass spectrometry (MALDI-TOF MS) (Bruker Daltronics), using the software MALDI Biotyper (MBT) Compass 4.1 with reference database MBT Compass Library DB-7854, according to the manufacturer’s instructions. Several species previously belonging to the *Clostridium* genus have undergone re-classification since January 2020 as defined in the database “List of Prokaryotic names with Standing in Nomenclature” (LPSN) as of 1 March 2022 [21]. These re-classified species were also included in this study since more changes of taxonomy are to be expected based on phylogenetic studies [22]. However, *C. difficile* was not included since the new name was already established in 2020. A MALDI Biotyper score of at least 2.0 was required for species determination, and if lower, the 16S rRNA gene was sequenced to assign the species [23]. Isolates only reported at the genus level after neither MALDI-TOF MS nor 16S rRNA methods successfully determined the species were named *Clostridium* spp. undefined in this study. The minimum inhibitory concentration (MIC) was determined using gradient strips (E-test, BioMérieux) on fastidious anaerobe agar (FAA, LabM) with the European Committee on Antimicrobial Susceptibility Testing (EUCAST) breakpoints for *C. perfringens* as of 2022 [24]. For the other clostridial species, no breakpoints have been established by EUCAST but guidance is provided for MICs, above which a suggestion is made to advice against the use of that agent [25]. Antibiotic susceptibility testing was not routinely performed for vancomycin or cephalosporins.

Polymicrobial bacteraemia was defined as growth of more than one bacterial species in the same blood culture (2 pairs of anaerobic + aerobic bottles). In cases where multiple clostridial species were identified in the same sample, the most frequently found species was defined as the main species and the least common one as co-pathogen.

### Underlying conditions and probable source of infection

Co-morbidities were evaluated using the modified Charlson Comorbidity Index (CCI) [26]. Immunosuppression was defined as an underlying diagnosis of immunodeficiency, any haematological malignancy, corticosteroid treatment (corresponding to ≥ 10 mg prednisolone for > 7 days), other immunosuppressive therapy or ongoing/recent (6 months) chemotherapy or stem cell transplantation within 12 months.

The source of infection was either based on the ICD-10 diagnose code or an obvious diagnosis based on review of the medical records, or otherwise recorded as “unknown”. An abdominal tumour was considered a probable source of infection if no other obvious source was present. A nosocomial infection was defined as the first positive blood culture drawn ≥ 48 h of hospital admission.

### Clinical and laboratory characteristics and outcome

All clinical characteristics were registered within 24 h of blood culture. Fever was defined as a temperature > 38.0 °C and hypotension as a mean arterial pressure ≤ 70 mmHg. Thrombocytopenia was defined below 150 × 10^9^/L, without previous known thrombocytopenia. Acute kidney failure was defined as a creatinine above reference, without previous known kidney failure. Sepsis and septic shock were defined according to the Sepsis-3 criteria [27]. Massive haemolysis was considered present when the laboratory reported that the sample could not be analysed due to haemolysis. Admission to the intensive care unit (ICU) was recorded within 10 days from the first positive blood culture. Treatment with a surgical procedure included all invasive therapeutic procedures performed as infection source control during the hospital stay after obtaining the first positive blood culture. All-cause mortality was recorded within 28 days from the first positive blood culture.

### Statistical analysis

The enumerator for incidence calculations was the number of unique infectious episodes, as defined above. The population denominator was defined as the year-end population with residency within the Skåne region, stratified by age and sex, retrieved from the database of Statistics Sweden, a governmental agency providing population statistics [28]. The age-standardised incidence rate was estimated by applying the direct standardisation method to the 2013 European standard population (in 5-year age strata, truncated at 90 +). Incidence rate ratios (IRR), including 95% confidence intervals, were used for comparisons across age and sex strata. Associations with 28-day mortality were evaluated using bivariate and multivariate logistic regressions, presenting odds ratios (OR) with 95% confidence intervals. In multivariate analysis, variables with a high degree of missingness (> 5%) were excluded, as were variables with suspected collinearity. To provide scale-independent comparability, ORs were presented for the 3rd quartile vs 1st quartile for continuous variables.

## Results

### Epidemiology

**Fig. 1:**
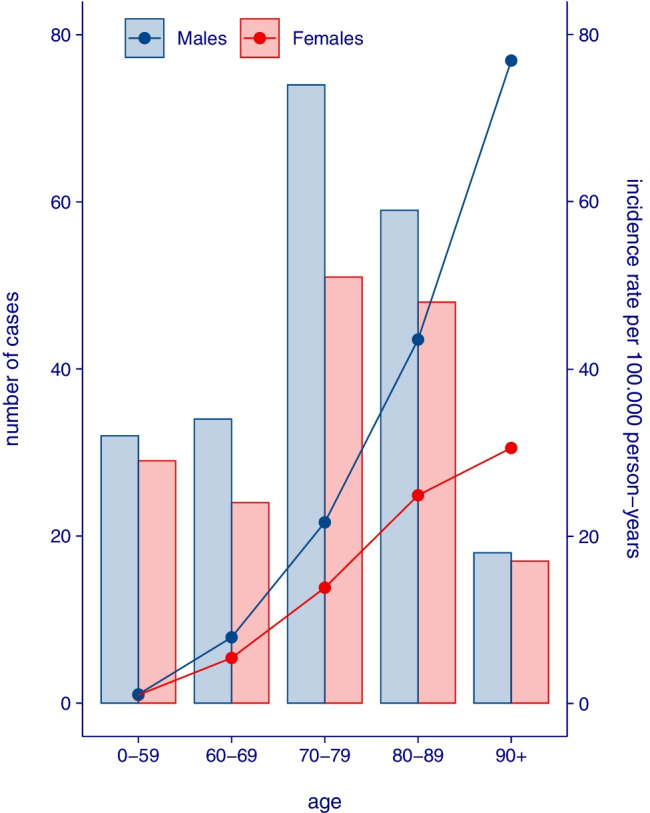
Number of cases and incidence rate of clostridial bacteraemia, by age and sex. Bars = number of cases (left y axis), dots and lines = incidence rate per 100.000 person-years (right y axis)

During the study period, 386 unique episodes with clostridial blood-stream infections were identified. The crude (95% CI) incidence rate was 4.8 (4.4–5.3) per 100.000 person-years and the age-standardised incidence rate was 4.9 (4.5–5.5). The median age was 76 years (IQR 66–83) and 217/386 (56%) of patients were males. There was a substantial age effect, with an IRR (95% CI) of 34.3 (25.5–46.6) for those aged 80 + vs 0–59, see Fig. 1. There was also a predominance for males in all age strata, with an overall crude incidence rate for males of 5.4 (4.8–6.2) per 100.000 person-years vs 4.2 (3.6–4.9) for females, resulting in an IRR (95% CI) of 1.29 (1.06–1.58). Incidence rates were stable over time during the 6-year period. When the 2022 taxonomy was used, the number of unique episodes was 322, resulting in an age-standardised incidence rate of 4.1 (3.7–4.6).

### Microbiology and species distribution

The most common species was *C. perfringens* accounting for 156/386 (40%) isolates, followed by *C. septicum* 52 (13%) and *C. ramosum* 48 (12%). The species distribution is presented in Table 1. Polymicrobial bacteraemia was present in 194/386 (50%) of cases, most commonly *Enterobacterales* (39%), followed by *Bacteroides* (14%) and *Enterococcus* (13%). In a majority, only one co-pathogen was present (124/194, 64%). Differentiating from the other species, bacteraemia with *C. septicum* was monomicrobial in 96% (50/52) and with *C. symbiosum* in 89% (8/9) of the cases.

**Table 1:** Species distribution of Clostridium* isolates

Species	*N* = 386 (%)
*Clostridium perfringens*	156 (40.4)
*Clostridium septicum*	52 (13.5)
*Clostridium ramosum*	48 (12.4)
*Clostridium innocuum*	20 (5.2)
*Clostridium clostridiiforme (Enterocloster clostridioformis)*	13 (3.4)
*Clostridium paraputrificum*	10 (2.6)
*Clostridium hathewayi (Hungatella hathewayi)*	9 (2.3)
*Clostridium symbiosum*	9 (2.3)
*Clostridium tertium*	8 (2.1)
*Clostridium cadaveris*	7 (1.8)
*Clostridium bolteae (Enterocloster bolteae)*	5 (1.3)
*Clostridium aldenense (Enterocloster aldenensis)*	4 (1)
*Clostridium citroniae (Enterocloster citroniae)*	4 (1)
*Clostridium sporogenes*	4 (1)
*Clostridium sordellii (Paeniclostridium sordellii)*	3 (0.8)
*Clostridium butyricum*	2 (0.5)
*Clostridium subterminale*	2 (0.5)
*Clostridium disporicum*	1 (0.3)
*Clostridium hylemonae*	1 (0.3)
*Clostridium indolis (Lacrimispora indolis)*	1 (0.3)
*Clostridium intestinale*	1 (0.3)
*Clostridium limosum (Hathewaya limosa)*	1 (0.3)
*Clostridium scindens*	1 (0.3)
*Clostridium* spp. undefined	24 (6.2)

^*^Species reclassified as not belonging to the *Clostridium* genus since January 2020 are included. New names in parenthesis

### Antibiotic susceptibility

**Fig. 2:**
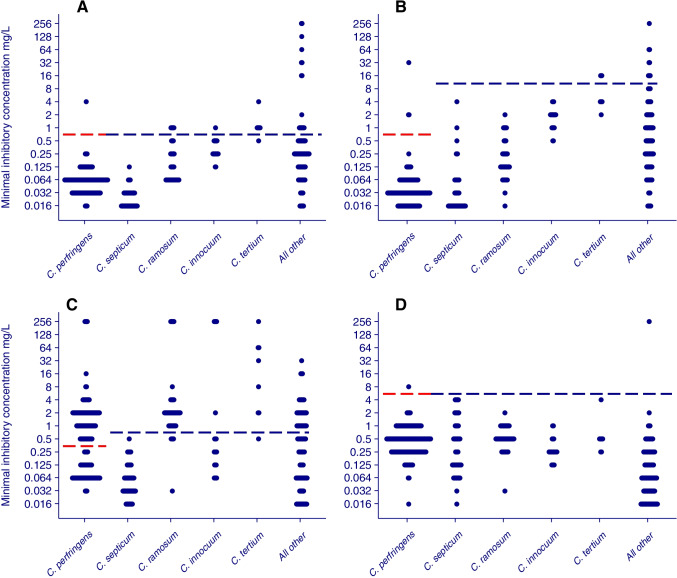
Minimum inhibitory concentrations (MICs) for the 4 most frequently found *Clostridium* isolates and for *C. tertium* due to its lower antibiotic susceptibility, by species and antimicrobial substance. Data are presented for penicillin G (**A**), piperacillin-tazobactam (**B**), clindamycin (**C**) and metronidazole (**D**). Each isolate is represented by a filled circle. SIR breakpoints according to EUCAST are indicated for *Clostridium perfringens* with R above and S below the red dashed line. No breakpoints have been established by EUCAST for the other clostridial species. Instead, cutoff values for these antibiotics have been suggested and if the MIC value is above the cutoff, advice against the use of the agent for treatment is given and the isolate can be regarded as resistant [25]. The cutoff values are indicated with blue dashed lines for the non-*perfringens* species

Using the recently published EUCAST MIC breakpoints for *C. perfringens*, all but 1, 1 and 3/156 *C. perfringens* isolates were susceptible to penicillin (MIC ≤ 0.5), metronidazole (MIC ≤ 4) and piperacillin/tazobactam (MIC ≤ 0.5), respectively, but 92/156 (59%) were resistant to clindamycin (MIC > 0.5). Due to the lack of clinical breakpoints for non-*perfringens* species, the frequency of susceptible isolates cannot be determined. In general, the MIC distributions varied considerably between species, but were almost universally low (MIC ≤ 4) for metronidazole. The MICs for all four antibiotics were also generally low (corresponding to S for *C. perfringens*) for *C. septicum* (Fig. 2) and MICs for carbapenems were low (≤ 0.125) for all species (not shown).

### Underlying conditions, clinical presentation and source of infection

The median CCI was 2 (IQR, 1–5). Of all patients, 84 (22%) had no underlying co-morbidities according to the CCI scoring system of which 34 (40%) had a diagnosis of either appendicitis or cholecystitis/cholangitis. *C. septicum* was unusual in the group without co-morbidities, accounting for only 4/84 (5%). Malignancy was the most common co-morbidity, in 182 (47%) patients, most pronounced for *C. septicum* in 40/52 cases (77%). Of the malignancies, haematological malignancies accounted for 26 (14%) of cases and 81 (45%) had metastatic disease.

Clinical information was available for 384 patients. Intraabdominal infection sites predominated where cholecystitis and cholangitis were the most common diagnoses in 49/384 cases (13%), caused by *C. perfringens* in 40/49 (82%). Soft tissue infections accounted for 25 (7%) of which two cases of necrotizing fasciitis and one case of Fournier’s gangrene were identified. No case of gas gangrene was identified (Table 2).

**Table 2:** Baseline characteristics, type of infection and probable source of infection for clostridial bacteraemia for the 8 most frequently isolated clostridial species

Baseline characteristics	All	*C. perfringens*	*C. septicum*	*C. ramosum*	*C. innocuum*	*C. clostridiiforme*	*C. paraputrificum*	*C. hathewayi*	*C. symbiosum*
n	386	156	52	48	20	13	10	9	9
Age, median (IQR)	76 (66–83)	77 (66–83)	77 (64–85)	75 (68–81)	73 (69–79)	75 (71–84)	78 (71–81)	80 (73–86)	62 (51–79)
Male sex	217 (56)	95 (61)	24 (46)	29 (60)	11 (55)	7 (54)	8 (80)	5 (56)	5 (56)
CCI, median (IQR)	2 (1–5)	2 (1–4)	3 (2–8)	2 (1–4)	2 (0–4)	2 (2–3)	4 (2–8)	1 (0–4)	2 (0–2)
Immunosuppression	96 (25)	37 (24)	15 (29)	13 (27)	7 (35)	5 (38)	1 (10)	0 (0)	1 (11)
Malignancy	182 (47)	63 (40)	40 (77)	20 (42)	9 (45)	7 (54)	5 (50)	3 (33)	4 (44)
Type of infection, n(%)									
Nosocomial	67 (17)	22 (14)	6 (12)	9 (19)	10 (50)	4 (31)	1 (10)	1 (11)	2 (22)
Polymicrobial	194 (50)	96 (62)	2 (4)	31 (65)	13 (65)	6 (46)	2 (20)	6 (67)	1 (11)
Probable source of infection, n (%)									
Appendicitis	21 (5)	2 (1)	1 (2)	5 (10)	2 (10)	1 (8)	0 (0)	2 (22)	4 (44)
Diverticulitis	17 (4)	5 (3)	0 (0)	1 (2)	2 (10)	2 (15)	1 (10)	1 (11)	1 (11)
GI tumour	46 (12)	14 (9)	18 (35)	3 (6)	0 (0)	1 (8)	2 (20)	0 (0)	0 (0)
Cholecystitis/cholangitis	49 (13)	40 (26)	0 (0)	3 (6)	0 (0)	0 (0)	0 (0)	1 (11)	0 (0)
GI perforation	41 (11)	13 (8)	7 (13)	7 (15)	3 (15)	4 (31)	0 (0)	2 (22)	0 (0)
Soft tissue/wound infection	25 (7)	6 (4)	2 (4)	4 (8)	0 (0)	0 (0)	0 (0)	0 (0)	2 (22)
Pancreatitis	3 (1)	3 (2)	0 (0)	0 (0)	0 (0)	0 (0)	0 (0)	0 (0)	0 (0)
Abdominal abscess	16 (4)	6 (4)	4 (8)	1 (2)	0 (0)	0 (0)	0 (0)	0 (0)	1 (11)
Unknown	127 (33)	50 (32)	17 (33)	17 (35)	10 (50)	5 (38)	4 (40)	2 (22)	1 (11)
Other	39 (10)	16 (10)	3 (6)	7 (15)	3 (15)	0 (0)	3 (30)	1 (11)	0 (0)

*IQR* interquartile range. *CCI* Charlson comorbidity index

In most cases, symptoms appeared within 24 h before the first blood cultures were obtained (median 0.5 days, IQR 0–2). In total, 54% of patients had fever, 37% had hypotension and 69% and 17% met the criteria of sepsis and septic shock, respectively (Table 3).

**Table 3:** Clinical presentation and outcome of clostridial bacteraemia

Clinical presentation	
Symptom duration days, median (IQR)	0.5 (0–2)
Fever (≥ 38.0 °C)	204/377 (54)
Hypotension (MAP < 70)	135/369 (37)
Sofa score, median (IQR)	3 (1–5)
Sepsis	264/384 (69)
Septic shock	64/384 (17)
Thrombocytopenia (< 150 × 10^9 / L)	110/326 (28)
Acute kidney failure	191/379 (50)
CRP, median (IQR)	102 (31.25–208.5)
Elevated lactate (> 2.0 mmol/L)	197/299 (66)
Outcomes	
Length-of-stay, median (IQR)	10 (5–18)
28-day mortality	100/384 (26)
ICU treatment	72/384 (19)
Vasopressors	64/72 (89)
Ventilator	43/72 (60)
Renal replacement therapy	18/72 (25)
28-day ICU mortality	33/72 (46)

*IQR* inter-quartile range, *MAP* mean arterial pressure. *ICU* intensive care unit

Massive haemolysis was present in 2 patients, both caused by *C. perfringens*, and death occurred within 48 h from obtaining the first positive blood culture.

### Treatment

Information on empiric antibiotic treatment was available for 385 patients of whom 158 (41%) received piperacillin/tazobactam, 114 (30%) received cefotaxime, in combination with metronidazole in 15 cases, and 51 (13%) received a carbapenem. Forty-two patients (11%) did not receive any empiric antibiotic treatment. Surgery was considered necessary as source control in 155 patients (40%), of whom 133 (35%) underwent surgical intervention. ICU treatment was given to 72 (19%) patients, see Table 3.

### Outcome

The median length of stay at the hospital was 10 days and the 28-day mortality rate was 26% (100/384). Sixty-seven of the 100 patients who died did not receive intensive care. Bivariate analysis revealed that age, absence of fever, high C-reactive protein (CRP) level on arrival, high SOFA-score, elevated lactate and the presence of sepsis or septic shock within 24 h from obtaining the first blood culture were all significantly associated with mortality. A similar pattern was seen in multivariate analysis (Table 4). Polymicrobial and monomicrobial infections differed slightly in clinical presentation and severity, but the difference in mortality was not statistically significant (Table 4 and Supplementary Table). The mortality in the group treated with cefotaxime only did not differ from overall mortality (24% vs 26%) (data not shown).

**Table 4:** Association between clinical factors and 28-day mortality

Factor	Died ≤ 28 days (*n* = 100)	Survived ≤ 28 days (*n* = 284)	OR (95% CI)*	p value	Multivariate OR (95%CI)*	Multivariate p value
Age	77 (69.8–85.2)	76 (65–82)	1.37 (1.02–1.85)	0.029	1.71 (1.16–2.53)	0.007
Male sex	51 (51)	165 (58)	0.75 (0.48–1.19)	0.219	0.62 (0.36–1.08)	0.091
Malignancy	53 (53)	128 (45)	1.37 (0.87–2.17)	0.172	1.67 (0.84–3.32)	0.147
CCI	2 (1–6)	2 (1–4)	1.21 (0.9–1.61)	0.209	1.3 (0.84–2.01)	0.245
Nosocomial infection	23 (23)	44 (15)	1.63 (0.93–2.87)	0.096	1.17 (0.59–2.32)	0.654
Polymicrobial infection	58 (58)	135 (48)	1.52 (0.96–2.42)	0.071	1.19 (0.69–2.06)	0.535
Immunosuppression	31 (31)	65 (23)	1.51 (0.91–2.51)	0.112	NA	NA
Absent fever	61 (65)	112 (40)	2.82 (1.74–4.59)	< 0.001	2.45 (1.42–4.2)	0.001
CRP	182 (92–246)	84 (24.8–182)	1.99 (1.42–2.77)	< 0.001	1.82 (1.24–2.67)	0.002
Sofa score	5 (2.5–8)	2 (1–4)	2.77 (2.03–3.77)	< 0.001	2.9 (2.01–4.17)	< 0.001
Sepsis	85 (86)	179 (63)	3.56 (1.93–6.58)	< 0.001	NA	NA
Septic shock	33 (33)	31 (11)	4.02 (2.3–7.03)	< 0.001	NA	NA
Lactate	5 (2.7–7.6)	2 (1.5–3.9)	2.16 (1.64–2.84)	< 0.001	NA	NA

Data are presented as n (%) for categorical variables and median (interquartile) for numerical variables. *OR* odds ratio (with 95% confidence interval). In the multivariate analysis, 376 observations (97.4%) were included. *OR for continuous variables are presented as 3rd quartile vs 1st quartile to facilitate scale-independent comparisons. *NA* variables not included in multivariate analysis, due to a high degree of missing data (lactate) or suspected collinearity

## Discussion

In this population-based observational study of clostridial bacteraemia, we present an incidence rate of 4.9/100.000 person-years. Patients were generally old with co-morbidities, especially malignancies, and a large proportion had a severe infection with acute onset, hypotension, organ failure and high fatality rate. Acute manifestations for severity of disease were associated with mortality whereas co-morbidities were not. Though massive haemolysis and severe necrotizing soft tissue infections are commonly described manifestations of clostridial infections in the literature, they were relatively uncommon in this study specifically addressing bacteraemia.

The existing studies on clostridial bacteriemia are mainly restricted to case reports, single-species studies, case series or single-centre studies, limiting the possibility to directly compare our incidence data with similar studies. However, an incidence rate of 1.8/100.000 person-years for clostridial bacteraemia was presented in a Canadian population-based study between 2000 and 2006 [8] and, in comparison, we present a more than two-fold higher incidence rate. Increased frequency of routinely drawn blood cultures or improved microbiological diagnostics could affect the incidence, but the similar mortality rates of 26% vs 30% in both studies indicate similar study populations. More advanced care in patients with high burden of disease may result in a true increase of incidence in accordance with several studies showing increasing incidence of bacteraemia and other infections requiring hospitalisation during the last 20 years [29, 30, 31, 32]. This also means that the generalizability of our results may not extend to younger populations especially in resource-poor areas.

Although population-based studies are lacking, making comparisons of absolute incidence rates between studies difficult, other findings can be compared. The highest incidence was seen in elderly patients, primarily in those over 80 years, consolidating the pre-existing data [8, 33]. A pronounced male gender predominance was noted in all age groups, similar to the population-based Canadian study [8], though it was not specifically commented in that study.

Concerning the microbiological characteristics, *C. perfringens* was the most frequently found species in accordance with previous research [1, 3, 4, 5] and polymicrobial growth was common, but interestingly, we found that *C. septicum* was an exception to this pattern with 4% polymicrobial growth only. To our knowledge, similar results have not been reported before and the clinical significance remains unclear.

As described in the literature, we found a clear association between clostridial bacteraemia and a known underlying malignancy, especially for *C. septicum* though no follow-up data on subsequent tumours for patients with no known tumour was available [9, 10, 11, 34]. The gastrointestinal tract dominated as the primary source of infection which is also reported previously [7, 33] and a pronounced predominance of *C. perfringens* in cholecystitis and cholangitis was noted in 82% of all the cases. Massive haemolysis due to *C. perfringens* was rare, but population-based or hospital-based numbers from other studies could not be found.

Our results tie well with previous studies wherein a low antibiotic resistance rate in *C. perfringens* has been observed [35, 36]. However, since the EUCAST MIC breakpoint for clindamycin was recently changed from 4 to 0.25, a large proportion of *C. perfringens* is now considered resistant, though the MIC distribution is unchanged. We observed higher overall MICs to penicillin and piperacillin/tazobactam in non-*perfringens* non*-septicum* clostridial species and MIC distributions to clindamycin that varied considerably between species, whereas MICs to metronidazole were universally low. Thus, our findings support avoiding the use of clindamycin as empiric monotherapy and addition of metronidazole may be a treatment option when *Clostridium* is suspected.

Patients in our study had acute onset of disease, short duration of symptoms and a high proportion of severe manifestations such as multi organ failure, hypotension and shock which strongly indicates that *Clostridium* is a significant finding in blood cultures and do not merely represent a transient bacteraemia, which is also supported by others [3, 18]. We found that all-cause 28-day mortality was mainly associated with factors that reflect the acute severity of disease but not with background factors such as co-morbidities or nosocomial acquisition. Hypothermia has previously been associated with mortality in clostridial bacteraemia [37] but these data were not available in our dataset. However, we found an association with absence of fever, and hypothermic patients may be within that group. Other studies have, depending on factors selected for analysis, found associations between mortality and increasing age, nosocomial acquisition, malignancy and other co-morbidities, length of hospitalisation, intensive care and surgical management [6, 19, 33, 34]. Patients with polymicrobial bacteraemia seemed to have a slightly more severe presentation. This could be due to the underlying source of infection, such as a perforation, or due to more virulent co-pathogens. However, there was no statistically significant effect on mortality. In the multi variate model, we did not include whether an efficient empiric antibiotic treatment was given. The reasons are several: Many infections were polymicrobial and we did not have antibiotic susceptibility data for all co-pathogens. Moreover, clostridia may be susceptible to cephalosporins (for an overview see [38]) but testing was not routinely performed, and, finally, we believe there may be an indication bias meaning that the more severely ill patients tend to get broad-spectrum antibiotics.

Our study has several limitations. First, retrieving retrospective data from medical records always includes missing data and a proportion of interpretation, in this study especially of complex information such as source of infection and the need for surgical intervention. Moreover, the residency status of patients was not retrieved, though the small proportion of residents seeking care outside the region and vice versa will only marginally affect incidence rates. Antibiotic susceptibility testing was performed by gradient strips which is not gold standard for anaerobic bacteria and should be interpreted with caution.

Future studies describing the incidence around the world are needed, and as the *Clostridium* genus is further genetically characterised to a less heterogenous classification, it should be possible to study clinical differences between clostridial species in more detail. Furthermore, though a clear association is shown between a known malignancy and *C. septicum* in particular, and *Clostridium* as a predictor for a subsequent colorectal cancer diagnosis has also been demonstrated for *C. septicum* and to less degree for *C. ramosum*, *tertium* and *perfringens* [11, 12], the sequence of events needs to be further investigated for other clostridial species to yield conclusive advice on screening in case of clostridial bacteraemia. Our study did not comprise data on later tumour diagnosis, and we plan a follow-up study on the incidence of subsequent tumours.

In conclusion, we present the highest incidence rate of clostridial bacteraemia reported to date. Clostridial bacteraemia is a severe condition affecting elderly with co-morbidities, most pronounced malignancies. The condition has high mortality that is associated mostly to the acute manifestations. Spectacular manifestations that render case reports such as gas gangrene, necrotizing fasciitis and intravascular haemolysis are relatively rare. Metronidazole is a universally efficient treatment option that could be added to broad-spectrum therapy when *Clostridium* is suspected. Until more data are available, in the light of the very high numbers of underlying malignancy, it seems reasonable that in clostridial bacteraemia without an obvious source of infection, work up for a gastrointestinal tumour should be considered.

## Supplementary information

Below is the link to the electronic supplementary material.

Supplementary file1 (DOCX 19 KB)

## Data Availability

The datasets generated during and analysed during the current study are not publicly available due to the risk of compromising individual privacy and due to the ethical permission. Upon reasonable request, the data can be made available only after a new application to the ethics review board.
