# Supplementary material for: Epidemiology, aetiology and clinical characteristics of clostridial bacteraemia: a 6-year population-based observational study of 386 patients
Source: Eur J Clin Microbiol Infect Dis. 2022 Sep 22;41(11):1305–14. doi: 10.1007/s10096-022-04491-8 (PMC9556422; doi:10.1007/s10096-022-04491-8)
Supplement: Supplementary file 1 — Supplementary file1 (DOCX 19 KB) [file 10096_2022_4491_MOESM1_ESM.docx]

**Supplementary Table**. Baseline characteristics, clinical characteristics and outcome compared between cases with monomicrobial and polymicrobial bacteraemia.

| **Baseline Characteristics** | **Monomicrobial** | **Polymicrobial** |
| --- | --- | --- |
| n | 194 | 192 |
| Age, median (IQR) | 76 (66-82) | 76 (66-83) |
| Male sex | 109 (56) | 108 (56) |
| CCI, median (IQR) | 2 (1-4) | 2 (1-5) |
| Immunosuppression. | 41 (21) | 55 (29) |
| Malignancy | 84 (43) | 98 (51) |
| **Type of infection, n(%)** |  |  |
| Nosocomial | 31 (16) | 36 (19) |
| Polymicrobial | 194 (100) | 0 (0) |
| **Probable source of infection, n(%)** |  |  |
| Appendicitis | 14 (7) | 7 (4) |
| Diverticulitis | 6 (3) | 11 (6) |
| GI tumour | 16 (8) | 30 (16) |
| Cholecystitis / cholangitis | 39 (20) | 10 (5) |
| GI perforation | 24 (12) | 17 (9) |
| Soft tissue / wound infection | 11 (6) | 14 (7) |
| Pancreatitis | 2 (1) | 1 (1) |
| Abdominal abscess | 6 (3) | 10 (5) |
| Unknown | 59 (31) | 68 (36) |
| Other | 16 (8) | 23 (12) |

| **Clinical presentation** | **Monomicrobial** | **Polymicrobial** |
| --- | --- | --- |
| Symptom duration days, median (IQR) | 0 (0-2) | 1 (0-3) |
| Fever (≥38.0°C) | 102 / 186 (55) | 102 / 191 (53) |
| Hypotension (MAP <70) | 56 / 183 (31) | 79 / 186 (42) |
| Sofa score, median (IQR) | 2 (1-4) | 3 (2-6) |
| Sepsis | 121 / 191 (63) | 143 / 193 (74) |
| Shock | 25 / 191 (13) | 39 / 193 (20) |
| Thrombocytopenia (<150 X 10^9 / L) | 46 / 159 (12) | 64 / 167 (17) |
| Acute kidney failure | 91 / 186 (49) | 100 / 193 (52) |
| C-reactive protein, median (IQR) | 102 (31-206) | 101 (34-224) |
| Elevated lactate (> 2.0 mmol/L) | 77 / 140 (55) | 120 / 159 (75) |
| **Outcomes** |  |  |
| Length-of-stay, median (IQR) | 10 (5-18) | 8 (4-17) |
| 28-day mortality | 42 / 191 (22) | 58 / 193 (30) |
| ICU treatment | 32 / 191 (17) | 40 / 193 (21) |
| Vasopressors | 24 / 32 (75) | 40 / 40 (100) |
| Ventilator | 19 / 32 (59) | 24 / 40 (60) |
| Renal replacement therapy | 6 / 32 (19) | 12 / 40 (30) |
| 28-day ICU mortality | 12 / 32 (38) | 21 / 40 (52) |
